# Couples Coping With Hematological Cancer: Support Within and Outside the Couple – Findings From a Qualitative Analysis of Dyadic Interviews

**DOI:** 10.3389/fpsyg.2022.855638

**Published:** 2022-05-19

**Authors:** Daniela Bodschwinna, Gregor Weissflog, Hartmut Döhner, Dietger Niederwieser, Anja Mehnert-Theuerkauf, Harald Gündel, Jochen Ernst, Ute Goerling, Klaus Hönig

**Affiliations:** ^1^Department of Psychosomatic Medicine and Psychotherapy, University Medical Center Ulm, Ulm, Germany; ^2^Comprehensive Cancer Center Ulm, University Medical Center Ulm, Ulm, Germany; ^3^Department of Medical Psychology and Medical Sociology, University of Leipzig Medical Center, Leipzig, Germany; ^4^Department of Internal Medicine III, University Hospital of Ulm, Ulm, Germany; ^5^Medical Clinic and Policlinic, Hematology, Cellular Therapy and Hemostaseology, Leipzig University Hospital, Leipzig, Germany; ^6^Charité – Universitätsmedizin Berlin, Corporate Member of Freie Universität Berlin, Humboldt-Universität zu Berlin, and Berlin Institute of Health, Charité Comprehensive Cancer Center, Berlin, Germany

**Keywords:** hematological cancer, couple (spouses), dyadic interview, individual coping, dyadic coping, social support

## Abstract

**Objective:**

Cancer affects the patients as well as their partners. Couples use different strategies to cope with cancer and the associated burden: individual coping, dyadic coping, and support from the social network and from professional health care. The aim of this qualitative dyadic interviews is to gain a deeper and more differentiated understanding of the support system inside and outside of the couple.

**Methods:**

Ten heterosexual couples (patients: seven men and three women) with different ages (patients: range = 22–75; spouses: range = 22–74), different hematological cancer (e.g., acute myeloid leukemia, non-Hodgkin’s lymphoma) and cancer stages (initial diagnosis or relapse) participated in the study. Semi-structured dyadic interviews were conducted. Data of the verbatim transcripts were systematically coded and analyzed following structuring content analysis.

**Results:**

Three main categories (individual coping, dyadic coping, and outside support) and ten subcategories about coping and support strategies in hematological cancer patients and their spouses could be identified. All couples described cohesion in relationship as an essential common dyadic coping strategy. Most strategies were focused on the patient’s wellbeing. Furthermore, couples reported different common plans for the future: while some wanted to return to normality, others were reaching out for new goals.

**Conclusion:**

Couples used various coping and support strategies, that differed in type and frequency between patients and spouses. Most of the strategies were perceived as beneficial, but some also triggered pressure. Overall, spouses seem to need more psychological support to improve their own wellbeing.

## Introduction

Hematological cancers include various heterogenous disease patterns that affect the hematopoietic system. Diseases like lymphoma, multiple myeloma and leukemia differ in treatment, progression, and forecast (Robert Koch Institute & Association of Population-Based Cancer Registries in Germany., 2021). Due to uncertainty about treatment effectiveness and cancer trajectory both patients and spouses suffer from psychological distress (Lambert et al., 2013; Kuba et al., 2019; Raphael et al., 2020). The couple faces new challenges (e.g., lack of knowledge about disease, financial burden), changing roles (e.g., single earner, family roles) and is concerned about their future together (Li et al., 2018; Serçe and Günüşen, 2021). In addition, highly aggressive treatments like high-dose chemotherapy, total body irradiation as well as treatment-related isolation of the patient in the case of stem cell transplantation leads to high burden in couples (Bishop et al., 2007; Beattie and Lebel, 2011).

Coping with cancer and its related burden can take place at different levels. Traditional models such as the transactional stress theory by Lazarus and Folkman (1984) focus on the individual centered view of stress management, in which coping efforts are described as an individual process. Over time dyadic approaches, in which couples respond to a shared stressor through a collaborative process, have gained increasing attention (Bodenmann, 1995). As cancer is a stressor that affects both patients and spouses as well as the close social network, coping and support efforts from all these parties should be considered (Bodenmann, 2005).

Individual coping, dyadic coping, and outside support are exceedingly important for couples facing cancer due to positive effects in adaption to the disease. Individual coping strategies such as reduction of negative mood, positive reappraisal or problem solving were generally associated with less psychological distress, fewer depression and anxiety symptoms (Brandão et al., 2017), higher quality of life, health and relationship satisfaction (Papp and Witt, 2010; Kvillemo and Bränström, 2014; Brandão et al., 2017). However, negative individual strategies like avoidance, denial, or resignation were related to higher psychological distress (Brandão et al., 2017). Dyadic coping strategies like open communication, positive and common dyadic coping were associated with improved relationship functioning and satisfaction, facilitation of couples’ intimacy, higher relationship quality, and fewer depression symptoms (Papp and Witt, 2010; Regan et al., 2014; Rottmann et al., 2015; Traa et al., 2015; Pankrath et al., 2018; Meier et al., 2019; Lupinacci et al., 2021; Ştefǎnuţ et al., 2021). In contrast to the positive effects, higher use of negative coping forms (e.g., avoidant coping), higher level of interference in regular activities, and perception of dyadic support behavior were associated with depression and anxiety in oneself (Lambert et al., 2013; Regan et al., 2014; Rottmann et al., 2015; Bodschwinna et al., 2021). Social support like support from friends, family, or colleagues usually has a buffering effect on depression, anxiety, and distress (Hasson-Ohayon et al., 2010; Fong et al., 2017; Lotfi-Jam et al., 2019; Bodschwinna et al., 2021), with the limitation that this may only be true if the support is wanted (Vodermaier and Linden, 2019). Regarding professional support, interventions appear to be beneficial for patients, spouses and couples and future direction points toward online interventions (Badr and Krebs, 2013; Badr et al., 2019; Luo et al., 2020).

A recent study supports the assumption that different coping strategies occur simultaneously and are used depending on availability (Paschali et al., 2021). This is in line with Bodenmann’s supplement of his sequential stress-coping-cascade model, which suggests that in continuous and cumulative stress situations people draw on different support simultaneously (Bodenmann, 1995, 2005). Furthermore, as individual and dyadic coping strategies are highly associated with each other (Papp and Witt, 2010; Paschali et al., 2021) it might be that combining specific forms of both strategies facilitate adaptation to cancer.

Despite the extensive quantitative research about coping with cancer, there have been fewer qualitative studies in this area. A recent interview study with hematological cancer patients and their family caregivers reported coping strategies like hiding emotions, thoughts and needs, a stronger dyadic relationship than in the past and changed roles within the dyad (Serçe and Günüşen, 2021). A systematic review of qualitative studies of men with prostate cancer identified coping strategies such as avoidance, employing positive focus, support seeking, retain pre-illness lifestyle and symptom management (Spendelow et al., 2018). However, less is known about the specific way in which different categories of coping strategies and support from outside of the couple are used by patients and their spouses as well as about the frequency with which they are used by each.

With the present interview study, we aim to explore how couples, in which one person is diagnosed with hematological cancer, cope individually and together with the disease and what coping and support strategies they use. Furthermore, we want to identify possible differences in coping behavior between the patient and partner. The deeper insights into specific types of coping and support strategies could improve the development of more detailed and tailored intervention programs for patients and their spouses suffering from cancer.

## Materials and Methods

Data of this qualitative study were collected as part of the project ‘Dyadic coping in hematological patients over time’ funded by the Deutsche José Carreras Leukämie-Stiftung between 2012 and 2015 (grant: DJCLS R 12/36). The corresponding multi-center longitudinal study examined a total of 330 couples at baseline and 217 couples at 6-month follow-up regarding their dyadic coping. Associations of dyadic coping with quality of life, relationship satisfaction, supportive care needs and psychological distress were investigated (Ernst et al., 2017; Weißflog et al., 2017; Pankrath et al., 2018; Bodschwinna et al., 2021). Since dyadic coping was a central aspect of the study, additional couple interviews were conducted to gain a deeper insight into the couples’ coping and support network. Ethics approval was obtained from the Ethics Committees of the Medical Faculty of the University of Leipzig (No. 298-12-24092012) and the University of Ulm (No. 243/12) and carried out in accordance with the Declaration of Helsinki.

### Participants and Procedures

Eligibility was based on the following criteria: Being a patient with hematologic cancer, living in partnership, age between 18 and 75 years, proficient in German. Both patients and partners were required to provide written informed consent prior to enrolment. The selection of couples for the interviews was made from a subsample of 100 couples early in the study process, who already finished the longitudinal survey. In order to reach a heterogeneous sample of couples with respect to age, gender, duration of relationship, type of hematological disease and total score of dyadic coping the maximum variation method was used (Moser and Korstjens, 2018). All couples were already made aware during participation in the longitudinal study that an interview request could be made afterward. Eligible couples for the interviews were contacted and informed about the procedure of the study via phone call and were invited to the clinic to a suitable appointment. In order to overcome challenging recruitment of couples (participation agreement of both), an expense allowance of 20€ per couple was provided. However, this did not significantly increase the willingness to participate.

### Data Collection

At baseline assessment of the longitudinal study sociodemographic information including sex, age, employment, education, marital status, living together, duration of relationship and medical information including diagnosis, disease type, disease status, and time since diagnosis were collected via paper–pencil questionnaire. In addition, dyadic coping was assessed with the Dyadic Coping Inventory (DCI) (Bodenmann, 2008). With 37 items, different aspects of dyadic coping are recorded and an overall sum score (range: 35–175) can be calculated. A higher score reflects more reported dyadic coping.

We conducted semi structured face-to-face couple interviews by one interviewer each (GW and DL) in Leipzig and Ulm between May 2014 and April 2015. The interviews were performed using a guideline prepared by the study team. The guideline consists of three thematic areas with open-ended questions: introductory question about the disease and its course, a question about how the couple talks about cancer and questions about what kind of support and coping is experienced within and outside the couple. The interview aimed to go beyond standardized questionnaires to gain a deeper insight into the couples’ coping system. A dyadic interview setting was used allowing participants to respond directly to each other’s statements, leading to more dynamism and more relevance to everyday life during the interviews (Froschauer and Lueger, 2003). The interviews were conducted in an undisturbed atmosphere, mostly in a clinic office and once in the couple’s home. All interviews were audio recorded, transcribed verbatim according to the predefined transcription rules and anonymized (Dresing et al., 2015).

### Data Analyses

Sociodemographic and medical information were reported with basic descriptive statistics using IBM SPSS Statistics 26. Total score of dyadic coping was calculated using sum score of all items of the DCI (Bodenmann, 2008). The transcripts of the interviews were analyzed using MAXQDA version 2020. The structuring qualitative content analysis by Kuckartz (2018) was applied. First step of this analysis process included the initial work with the interviews, where important text passages were highlighted. Within the second step, main categories were developed deductively according to the basic theory. The third step included a first coding of all interviews using the main categories. In the fourth step all these passages were compiled per main category. Based on these collected text passages, subcategories were inductively formed in the fifth step. Since the subcategories of dyadic coping and outside support already exist theoretically, their configuration was not purely inductive. The remaining subcategories were formed inductively on basis of the interviews conducted. All these subcategories could be confirmed by the interviews. The resulting category system was discussed within the research team, adjusted twice through back-and-forth comparison with literature and interview content. Afterward an associated coding guide was developed with definitions, anchor examples of each subcategory and coding rules. The sixth step included coding of the entire material with this category system. Within the seventh and last step each subcategory was analyzed thematically and presented in summary (Kuckartz, 2018). The coding steps three and six were conducted by two researchers (DB and UG) independently. Inconsistent coding decisions were discussed by the coders to reach consensus. Interrater agreement was calculated with Kappa according to Brennan and Prediger (1981) and amounts κ_n_ = 0.77.

## Results

### Sample Characteristics

Of 35 couples approached, 25 couples (71.4%) declined to participate, either by both partners or by one partner. The final sample comprised 10 heterosexual couples (seven male and three female patients). Since no substantial new information was obtained after the 10 interviews, data saturation could reasonably be assumed and re-recruitment was declined. The mean age in patients was 57.0 years (SD = 16.09, range = 22–75) and in partners 54.3 years (SD = 17.68, range = 22–74). The duration of relationship ranged between 2 and 52 years. Five patients had acute leukemia, three had non-Hodgkin lymphoma, and one each had multiple myeloma and chronic leukemia. The majority of patients were either in full remission (*N* = 5) or partial remission (*N* = 2) and time since diagnosis was less than 2 years for seven patients. The interviews lasted between 47 and 116 min. Sociodemographic and medical characteristics are given in Table 1. Structuring content analysis resulted into three main categories: individual coping, dyadic coping, and outside support, each with several associated subcategories. Table 2 shows the identified categories as well as the proportions of coping and support strategies used by patient and the spouse.

**TABLE 1:** Patient and spouse characteristics.

Characteristics		Patient		Spouse
				
		*N*		*N*
Sex	Male	7		3
	Female	3		7
Age mean (SD, range)		57.0 (16.1, 22–75)		54.3 (17.7, 22–74)
Employment	Pension/early retirement	7		4
	Employed	2		5
	Unemployed	1		1
Education	<10 years	2		1
	10 years	3		6
	>10 years (High school)	5		3
Total dyadic coping mean (SD, range)		131.8 (20.3, 89–155)		127.5 (15.1, 108–152)
			
			Couples	
			
			*N*	
			
Marital status	Married		8	
	Not married		2	
Living together	In same household		9	
	In separate households		1	
Duration of relationship – years, mean (SD, range)			24.4 (21.6, 2–52)	
		
		Patient		
		
		N		
		
Diagnosis	Acute leukemia	5		
	Chronic leukemia	1		
	Non-Hodgkin	3		
	Multiple myeloma	1		
Disease type	Initial diagnosis	8		
	Relapse	2		
Disease status	Full remission	5		
	Partial remission	2		
	Not assessable	3		
Time since diagnosis	≤2 years	7		
	3–5 years	2		
	>5 years	1		

**TABLE 2:** Patients’ and spouses’ quotes and frequency of categories identified.

Categories and subcategories	Number of interviews (*n* = 10)	Number of total text passages (*n* = 520)	Patients’ text passages (*n* = 272)	Spouses’ text passages (*n* = 248)	Representative quotes
**Individual coping**					
Emotional focused	10	79	56 (70.9%)	23 (29.1%)	“You have to do something. I can’t sit around somewhere and lie and think about it and do nothing. I can’t do that. So, I have to get out as much as I can. Even into the woods or whatever. I did wood, I did the horses. Everything I could do, I did. I did the garden, I planted hedges. So just those things.” [P7]
Problem focused	10	72	44 (61.1%)	28 (38.9%)	“But I think I have started making lists right from the day of diagnosis: I have to resolve all this, and I have to do all this.” [S3]
Positive reframing	9	60	31 (51.7%)	29 (48.3%)	“And the confidence that I have always shown. Just the diagnosis didn’t cause hysteria in me, in any way. So, I’m not jumpy around and now I have to make a will and this and that. Nothing like that. It was simply: We can do it together! […] That is a danger, but not the end. And that’s actually what kept us going” [P9]
**Dyadic coping**					
Stress communication	8	40	30 (75.0%)	10 (25.0%)	“We have actually addressed everything, as said whether positive or negative.” [P4] “Yes, we actually talked through all facets of the disease in our minds. Always together. As hard as it may be.” [S4]
Supportive dyadic coping	9	41	7 (17.1%)	34 (82.9%)	“So, I was there every day. I went to work, then I went home and organized everything, cooked him something and then stayed as long as I could.” [S1]
Delegated dyadic coping	7	16	0	16 (100%)	“I then took over that at home. Washing clothes, cleaning, and shopping and so on.” [S6]
Negative dyadic coping	4	10	4 (40.0%)	6 (60.0%)	“So, he meant many things well. But I have also felt under pressure from time to time. And he didn’t realize that. Because he had such stress and then he said: ‘Do this, do that.’ And it always had to be done immediately.” [P10]
Common dyadic coping	10	110	38 (34.5%)	72 (65.5%)	“Of course, I would say that we got through it quite well and stuck together.” [S3] *-cohesion* “It was important to me that everything is done at home as we are used to it, in this case. There was a bit of a handicap, but we have actually maintained the daily routine.” [P9] *– return to normality* “But you should also turn to new things every now and then. We try to practice that now always?” [P6] *– new goals*
**Outside support**					
Social Support	10	50	42 (84.0%)	8 (16.0%)	“They [family and friends] can’t help you either, but they can give you moral support. And they have supported. They came then. They always asked when we could visit [the patient] and they did, even if it was only for 10 min but at least they visited [the patient].” [S6]
Professional support	8	42	20 (47.6%)	22 (52.4%)	“But I’m glad when I have my doctor. Someone who understands me. Someone who says, yes, you have a hard time at the moment, but you can do it and you’ll get out of it.” [P5]

*S = Spouse, P = Patient.*

### Individual Coping

#### Emotional Focused Strategies

Patients reported a variety of activities they undertook to improve their emotional wellbeing and relieve their stress, such as exercise, walking, reading books, playing games, watching movies, writing, practice rituals, meditation and relaxation techniques. In addition, some enjoyed trivia like trying to keep one’s sense of humor or enjoyed motivational sayings. Acceptance of the disease and its consequences was perceived to promote serenity thus fostering calmness and relaxation. The spouses reported overall fewer strategies for improving their wellbeing. They also done some exercise, but indicated more emotional strategies such as rumination, distraction, crying or feelings of anger and helplessness while staying home alone during the hospitalization period of the patient.

#### Problem Focused Strategies

Among patients, problem focused strategies were characterized by seeking information about treatment and medicine and engaging in health-promoting activities. Moreover, prioritizing by importance to focus on what is most essential and being realistic overall were also mentioned as problem focused strategies. In contrast, some also wanted to cope with problem avoidance through suppression and downplaying. Some spouses also searched for information, created lists for their tasks, planed the way back into everyday life, set boundaries and reduced extra work to have more time for themselves and the patient.

#### Positive Reframing

In all couples except one, patients and spouses reported different kinds of positive attitude and positive reframing. Positive reframing showed itself in hope and confidence, encouraging yourself, a new courage to live in patients and gratitude even for small advances. A wide variety of plans for the future reflected an optimistic outlook, both among patients and spouses.

### Dyadic Coping

#### Stress Communication

While at the beginning of the disease there was a daily exchange about the disease and treatment, over time the disease fades into the background in communication. Some couples described an open communication, where they talked about everything, all facets of the disease, both positive and negative. Most of the stress communication, including fear and frustration, came from the patient, who reported that it is relaxing to have someone to communicate with. Spouses, on the other hand, who are often the first point of contact for any problems, reported that the patient’s stress communication is very exhausting, but often this was the only way for the patient to relieve stress.

#### Supportive Dyadic Coping

Most supportive dyadic coping was provided by the spouses. One major part of this was simply being there for the patient including almost daily visits in hospital. Other spousal support includes instrumental, emotional and informational support, such as driving to appointments, physical closeness, providing distraction and encouragement, and gathering information about the disease and related issues. Not being alone and the presence of someone who is interested in the patients’ sensitivities was perceived as very important by the patients, and for the spouses the feeling of being able to do something was also important. For their part, the patients were also concerned about their spouses and tried to support them again more over time.

#### Delegated Dyadic Coping

Only spouses reported temporarily taking over of certain tasks to relieve the patient’s burden. This comprised instrumental support such as daily household tasks, childcare and organization of paperwork associated with the disease and sick leave, but also continuing of patients’ leisure activities until they can take them over again. In addition, the spouses provided informational support by obtaining information from physicians or internet research. Patients appreciated this support as extremely helpful. One spouse reported the fear of being seen only as a caregiver and no longer as an equal partner.

#### Negative Dyadic Coping

Both patients and spouses indicated types of negative dyadic coping. Two patients reported individual situations in which the spouse does not take seriously their stress and alternative coping attempts. Moreover, some advice from spouses were perceived as triggering pressure by patients. Similarly, spouses reported conflict and feeling rejected when some of their support efforts were described as exaggerated and unhelpful by the patients. Mentioned examples of mutual negative dyadic coping were wordy disputes and not considering the other’s point of view.

#### Common Dyadic Coping

Across couples, common dyadic coping was most frequently mentioned. Patients and spouses described multiple topics within common dyadic coping. The cohesion in the partnership, getting through the illness together and being there for each other were reported by all couples. These actions are associated with the deeper feeling of “we” and the strengthening of relationship. Some couples share that they show understanding of each other’s feelings and regulate intense emotions together. There was also joint problem solving and joint decision-making regarding treatment and related areas (e.g., joint meal changes). Additionally, couples demarcate themselves together by taking distance from negative things and people or by relinquishing burdensome as well as time-consuming things. Moreover, while some couples reported that they try to live a normal everyday life again after the disease moved into the background, other couples used it as opportunity for new joint ventures, hobbies, and interests.

### Outside Support

#### Social Support

Family and friends provided emotional support through visits and contact via telephone. They inquired about the patient’s condition, while the partner tended to take a back seat. Partly the contact decreased over time, especially when they were afraid to talk about the disease. Exchange with other patients from the circle of friends was more open-minded and intensive. Family and friends also provided helpful instrumental support and friends with medical background helped with information and explanations. However, patients also felt pressured by some recommendations from friends. Various joint activities and spending time with friends and family provided joy and distraction for the couple. Only one spouse reported that a relative directly took care about the spouses’ condition and advised a break. Another important factor was the support from the boss, who allowed to vary the working hours for the spouse to get more flexibility.

#### Professional Support

Both patients and spouses used professional offers for support services. Spouses emphasized the importance of supervision by a psychotherapist and the exchange with this neutral person. Patients perceived the assistance from social workers in making applications and referrals to other services as very helpful. Understanding and moral support from friendly, patient, and well-trained physicians and nurses, who treated the patient as a human being, was also reported by patients. Overall, many would recommend psychological support, especially for the partner. The wish for more proactive offers from the clinical side was expressed.

## Discussion

Coping and support strategies are key factors in improving wellbeing and adjustment in couples facing cancer. The purpose of this qualitative study was to go beyond quantitative data from prior questionnaire studies and to gain deeper insight into the specific strategies that are summarized in the theoretical categories. Compared to our previous quantitative studies (Ernst et al., 2017; Weißflog et al., 2017; Bodschwinna et al., 2021), we were able to confirm the main coping and support categories, as well as show similarities in the distribution of coping and support proportions between patient and partner. In addition, with the deeper insight new distinctions within these categories could be identified as well as the extensive scope of some categories. To our knowledge, this is the first study considering different coping and support strategies in cancer patients and their spouses using a dyadic interview setting.

Not only the frequency of individual coping strategies reported within a subcategory but also the type of strategy differed between patients and spouses. Regarding emotional strategies, patients focused on a variety of activities improving emotional wellbeing, while spouses reported more unfavorably strategies such as rumination or distraction. This is in line with previous research indicating that spouses commonly neglect their own need regarding wellbeing and are always with the patient in thought (Heynsbergh et al., 2019). Differences in problem focused strategies may be explained by role: patients reported more strategies which are directly associated with the diagnosis, while spouses focused more on tasks around and duties to path the way back to everyday life. The realistic approach of patients to the disease has already been reported as a strategy for advanced cancer patients (Walshe et al., 2017). The high level of positive reframing and future plans reported in both patients and spouses may be due to the fact that the majority of patients were in remission and therefore in a positive mood overall. On the other hand, this high level of positivity could be also due to patients’ misunderstanding of their prognosis, which seems to be very common in palliative patients (Jacobsen et al., 2013). Positive reframing in our study was also expressed through gratitude and enjoyment of little things, which is a way of living in the now that has also proven important for patients with advanced cancer (Cottingham et al., 2018).

Open communication is essential for couples coping with cancer (Goldsmith and Miller, 2014; Li et al., 2018), but due to individual differences in couples, research should avoid the general and abstract concept of openness and move on to more differentiated descriptions (Goldsmith and Miller, 2014). As in previous dyadic studies, we found that communication within the dyad can change over cancer trajectory (Siminoff et al., 2020). Communicating stress in particular is positively related to better relationship quality, facilitates couples’ intimacy and reduced distress (Badr et al., 2018; Lupinacci et al., 2021; Ştefǎnuţ et al., 2021). The relaxing effect of stress communication was also reported by patients in our study, while some spouses complained about how burdensome patients’ stress communication was for them. To address these differences, couple-based interventions should change the generic notion of open communication into more individual approaches (Badr, 2017).

Because of the role-effect it is not surprising, that spouses provided more supportive dyadic coping than patients (Kroemeke et al., 2019). Spousal support was especially strong in the post-diagnosis period and during hospitalization (Antoine et al., 2013), while patients tried to reestablish support after recovery and return home (Kroemeke et al., 2019). Being there for the patient was a salient issue for the couple, helping both not to feel alone. Besides supportive behavior, spouses also took over tasks completely when the patients were unable to do so (Palmer Kelly et al., 2019). Thus, spouses may also worry about being perceived as caregivers only which may turn the relationship out of balance (Serçe and Günüşen, 2021).

The overall less reported negative dyadic coping may likely be due to a selection bias given that well-functioning couples might have been more willing to be interviewed. The fact that the interview was conducted jointly with both partners might additionally have reduced the chance to observe negative dyadic coping. Apart from this reasoning well-intentioned support from spouses can also cause negative acknowledgment from patients, if support does not match patient’s needs (Palmer Kelly et al., 2019).

Within common dyadic coping, which was the most frequent coping strategy in our sample, cohesion in relationship was important in all couples and manifested in various manner. In addition to joint activities, it also seems to be essential to jointly distance oneself from negative and stressful things. Through all these experiences, couples achieve mutual growth, more intimacy and improvement in their relationship (Beattie and Lebel, 2011; Hasson-Ohayon et al., 2016; Lupinacci et al., 2021; Serçe and Günüşen, 2021). In addition, the couples could be divided into two groups: those who wanted to return to normality of the everyday life (Antoine et al., 2013) and those who wanted to discover new things together. This distinction in different future plans was also partially reflected in the individual coping strategies.

Like in our previous quantitative study as well as in other studies, spouses in the present sample reported less direct social support from friends and family compared with patients (Hasson-Ohayon et al., 2010; Bodschwinna et al., 2021). Nevertheless, instrumental social support for the patient can also be indirectly relieving for the spouse. Support from friends and family is perceived as helpful by most patients, while some recommendations cause patients to feel pressure. Therefore, it seems that social support should also meet the expectations or needs of patients in order to be beneficial (Reynolds and Perrin, 2004; Vodermaier and Linden, 2019). Over time social networks changed and received social support decreased for some of the participants, which can be due to burnout of support providers or because patients no longer needed outside support (Arora et al., 2007; Palmer Kelly et al., 2019). Professional support is widely used in couples. Spouses in our sample were more likely to go to psychotherapist, while patients reported more instrumental help from social workers. More proactive support offers in general and psychological support for the spouses were desired, as perception of available support is low (Li et al., 2018; Heynsbergh et al., 2019).

## Study Strength and Limitations

The combination of deductive and inductive categorization strategies is clearly the strength of the present qualitative study. This approach is well in line with previous research and theories, and additionally allows for more flexibility in identifying additional subcategories. Furthermore, the dyadic interview setting has the advantage to explore coping and support strategies of the couple with both partners together, since they also concern both of them. Some limitations of the study should be considered. Given that both partners had to agree to participate, there may be a selection bias in the sample toward couples who are in highly functional relationships and tend to communicate more openly. Furthermore, the high dropout rate is to be considered as a limitation. The reasons for this probably lie in the logistical challenge for the couples to appear together for an on-site appointment. Due to the large catchment area of the clinics, long distances often also had to be covered. Another limitation is, that the assumption of theoretical data saturation after 10 interviews did not follow the typical process. Initially, an unexpected high percentage of the couples approached declined to participate. Only subsequently re-recruitment was declined, as a review of the processed interviews revealed that no significant new information had been added in the most recent interviews. The ineffectiveness of the expense allowance may be due to the fact that a financial incentive is not a consideration for couples facing life-threatening cancer. Conducting the interview together may also have resulted in social desirability influencing the two partners’ communication. While an effort was made to include heterogenous couples, some important variables were not considered. Therefore, our sample consisted mainly of patients in remission and the currently attenuated symptoms could account for the low proportion of negative coping strategies reported. Furthermore, generalization of our findings to other cancer types is not applicable, as this study was conducted only with hematological cancer patients and their partners.

## Clinical Implication

Couples facing cancer use a variety of different coping and support strategies. In this context, patients and spouses differ in some of their used strategies and received support. Clinicians should keep track of the strategies used, intervene when they prove not to be useful, and recommend tailored improvement of the strategies. First, open communication should not be generally recommended, as stress communication has been shown to be both beneficial and burdensome (Badr, 2017). Furthermore, it should be highlighted, that we could identify two different types of future plans in couples: returning to normality and reaching out for new goals. These new insights could serve as a new direction for couple interventions by adding tools that help realizing the couples’ individual plan for the future. Tailored support in couple-based online interventions could be a suitable implementation for this purpose (Luo et al., 2020). In addition, health care system should provide more proactive offers for psychosocial support for spouses, as their suffering still seems to be rather neglected. Emotional coping, in particular, should be improved in spouses, as they used very few strategies to relax and to improve their own wellbeing. Especially during the patients’ hospitalization, clinicians should observe spouses’ condition and recommend appropriate support services.

## Conclusion

We identified various coping and support strategies regarding individual coping, dyadic coping, and outside support. Most of them were perceived as beneficial, but some triggered pressure. With qualitative research we were able to get a more detailed and deeper insight into the different strategies. For example, common dyadic coping showed various facets that a representation with a numerical value could not do justice to. We were also able to identify some differences in patient and spouse strategies that should be considered in couple intervention development. Further research in the area of coping and support strategies could gain even deeper insights by examining real-time interactions between patients and spouses (Lau et al., 2019).

## Data Availability Statement

Data are only available from the corresponding author on request, because of privacy or ethical restrictions.

## Ethics Statement

The studies involving human participants were reviewed and approved by Ethics Committees of the Medical Faculty of the University of Leipzig (No. 298-12-24092012) and the University of Ulm (No. 243/12). The patients/participants provided their written informed consent to participate in this study.

## Author Contributions

JE, GW, KH, and HG were responsible for funding and design of the study. AM-T, DN, and HD supervised design and conduction of the study in terms of psychological or hematological expertise. GW, JE, KH, and DB were responsible for recruiting participating couples and conducting the interviews. DB was responsible for the evaluation method and the development of the code system, performed the first coding of the interviews, supervised and contributed to the qualitative data analysis and interpretation, performed the quantitative analysis, and drafted the manuscript. UG was responsible for second coding of the interviews. All authors have read the manuscript critically and approved the final manuscript.

## Conflict of Interest

The authors declare that the research was conducted in the absence of any commercial or financial relationships that could be construed as a potential conflict of interest.

## Publisher’s Note

All claims expressed in this article are solely those of the authors and do not necessarily represent those of their affiliated organizations, or those of the publisher, the editors and the reviewers. Any product that may be evaluated in this article, or claim that may be made by its manufacturer, is not guaranteed or endorsed by the publisher.
