# Supplementary material for: Couples Coping With Hematological Cancer: Support Within and Outside the Couple – Findings From a Qualitative Analysis of Dyadic Interviews
Source: Front Psychol. 2022 May 19;13:855638. doi: 10.3389/fpsyg.2022.855638 (PMC9161167; doi:10.3389/fpsyg.2022.855638)
Supplement: Supplementary file 1 [file Data_Sheet_1.PDF]

Introduction:

1. First of all, I would like to ask you to look back over the last few months. What has happened in this time in relation to your disease, what was important for you, what was rather unimportant?
2. Could you tell me something about the course of the disease, e.g. when the suspicion arose and when the diagnosis was made?
    a. If the partner does not enter into the conversation of his/her own accord, he/she should be encouraged to do so: e.g. "How did you as a partner perceive it?”

Communication of the couple:

3. Could you describe to me how you as a couple exchanged information about the disease after the diagnosis?
    a. If the partner does not enter into the conversation of his/her own accord, he/she should be encouraged to do so

Support and coping inside and outside the couple:

4. How did you deal with the disease? What support did you receive? What was that like for you? Could you please tell us how that went?
5. Often, not only in the case of serious illnesses, the partners are a support for the sick person. Sometimes, however, the partners themselves are under so much strain that they cannot cope with the situation. How is it with you? In which situation or phase of illness were you particularly supported by your partner, and in which less so? Which other support do receive?
6. When you reflect on the topics discussed so far, especially about coping with the disease, what has been particularly helpful to you alone or as a couple? What was difficult?
